# Incidence and Factors Associated With Development of Fistula-in-Ano After Drainage of Anorectal Abscess: A Prospective Observational Study

**DOI:** 10.7759/cureus.114799

**Published:** 2026-08-19

**Authors:** Pankaja S. S., Winthrop J Pereira, Thrishuli P. B.

**Affiliations:** 1 General Surgery, JSS Academy of Higher Education and Research, Mysuru, IND

**Keywords:** anorectal abscess, anorectal sepsis, cryptoglandular infection, fistula-in-ano, follow-up, incision and drainage, risk factors

## Abstract

Background

Anorectal abscess and fistula-in-ano represent acute and chronic manifestations of cryptoglandular anorectal sepsis. Fistula formation after incision and drainage remains an important cause of morbidity and recurrent sepsis. Evidence regarding demographic, socioeconomic, dietary, hydration, bowel habit, and clinical factors associated with fistula formation is limited, particularly in the Indian population. This study assessed the six-month incidence of fistula-in-ano following abscess drainage and factors associated with its development.

Methods

A prospective observational study was conducted among 65 adults undergoing incision and drainage of anorectal abscess at JSS Medical College and Hospital, Karnataka, India. Patients were followed for six months. Demographic, socioeconomic, comorbidity, dietary, lifestyle, bowel habit, abscess, and microbiological variables were recorded. Associations with fistula formation were assessed using Pearson chi-square, Fisher’s exact, or Fisher-Freeman-Halton exact tests, as appropriate. Significant variables were further assessed using univariate binary logistic regression. Odds ratios (ORs) with 95% confidence intervals (CIs) were calculated. A two-sided p <0.05 was considered significant.

Results

Fistula-in-ano developed in 22/65 patients (33.8%); of the remaining, 39 (60.0%) achieved complete healing without fistula and four (6.2%) developed recurrent abscess without fistula. Male sex (OR 7.20, 95% CI 1.36-38.20; p=0.014), age 61-70 years (OR 6.60, 95% CI 1.23-35.44; p=0.028), abscess size >3 cm (OR 6.88, 95% CI 1.98-23.88; p=0.002), mixed diet (OR 2.99, 95% CI 1.03-8.66; p=0.043), constipation (OR 6.64, 95% CI 1.71-25.76; p=0.006), diabetes mellitus (OR 4.28, 95% CI 1.33-13.75; p=0.014), and water intake <1.5 L/day (OR 3.40, 95% CI 1.01-11.45; p=0.048) were associated with higher odds of fistula formation. Abscess type was significantly associated with fistula formation (p=0.007). After recoding, patients with ischiorectal, submucosal, or supralevator abscesses had higher odds than perianal abscesses (OR 19.20, 95% CI 2.06-179.08; p=0.009). Lower socioeconomic status was significantly associated with fistula formation on Fisher-Freeman-Halton exact testing (p=0.003) but was not included in logistic regression because no fistula events occurred in the upper socioeconomic group, resulting in complete separation and unstable parameter estimates. Abscess position was significant on categorical analysis (p=0.013), although posterior location was not significant on regression (p=0.113). Among the 22 patients who developed fistula-in-ano, 13 (59.1%) had posterior internal openings and 16 (72.7%) had intersphincteric fistulas.

Conclusion

One-third of patients developed fistula-in-ano within six months following drainage of anorectal abscess. Male sex, older age, larger abscesses, diabetes, constipation, mixed dietary pattern, low water intake, and non-perianal abscess type were associated with increased odds of fistula formation. These findings identify potentially high-risk clinical and modifiable characteristics that may help guide closer follow-up and patient counselling after abscess drainage. However, the associations should not be interpreted as independent predictors because multivariable analysis was not performed. Larger multicentre prospective studies with longer follow-up and multivariable modelling are required to validate these findings and establish independent risk factors.

## Introduction

Anorectal abscess is an acute manifestation of anorectal sepsis, most commonly resulting from infection and obstruction of an anal gland, whereas fistula-in-ano represents the chronic consequence of persistent infection and communication between the anal canal and perianal skin. The cryptoglandular theory remains the principal explanation for the development of most idiopathic anorectal abscesses and fistulas. Obstruction of an anal gland may result in infection within the intersphincteric plane, with subsequent extension into surrounding tissues producing an abscess; persistence of the infected gland or epithelialised communication with the anal canal may subsequently result in fistula formation [1,2].

Prompt incision and drainage remains the cornerstone of treatment for an anorectal abscess. Despite adequate drainage, a proportion of patients subsequently develop fistula-in-ano or recurrent abscess. Published studies have reported widely varying rates of fistula formation, partly because of differences in patient selection, abscess characteristics, follow-up duration, and methods of outcome ascertainment. Previous studies have reported fistula formation rates of approximately one-third or more following abscess drainage [3-6].

Fistula-in-ano is commonly classified according to the Parks classification, which categorises fistulas into four major anatomical types based on their relationship to the anal sphincter complex: intersphincteric, transsphincteric, suprasphincteric, and extrasphincteric. The Parks classification remains the most widely accepted anatomical classification because it correlates closely with the path of the fistulous tract and assists in selecting the most appropriate surgical management. Intersphincteric fistulas are the most common subtype, whereas suprasphincteric and extrasphincteric fistulas are relatively uncommon and usually represent more complex disease [2].

Several demographic and anatomical factors have been investigated as potential predictors of fistula formation, including age, sex, abscess location, abscess size, smoking, diabetes mellitus, and microbiological characteristics. However, evidence regarding lifestyle factors such as hydration, constipation, dietary pattern, and socioeconomic status remains limited, particularly in the Indian population [3-6]. The association of these potentially modifiable factors with fistula development following abscess drainage has not been adequately explored in prospective studies.

Identification of patients at increased risk of fistula formation is clinically important because it may facilitate closer postoperative surveillance, earlier diagnosis, timely intervention, and improved patient counselling. Better understanding of these factors may also contribute to optimisation of postoperative management and follow-up strategies. Therefore, the present prospective observational study was undertaken to determine the six-month incidence of fistula-in-ano following incision and drainage of anorectal abscess and to evaluate the association of demographic characteristics, socioeconomic status, comorbidities, dietary and lifestyle factors, bowel habits, abscess characteristics, microbiological findings, and perioperative variables with subsequent fistula formation in patients treated at a tertiary care teaching hospital.

## Materials and methods

Study design and setting

This prospective observational study was conducted in the Department of General Surgery at JSS Medical College and Hospital, JSS Academy of Higher Education and Research, Mysuru, Karnataka, India, over a period of 18 months from November 2023 to May 2025. The study was approved by the Institutional Ethics Committee (approval number: JSS/MC/PG/2324/2023-24), and written informed consent was obtained from all participants before enrolment. 

Study population

Adult patients (≥18 years) presenting with a primary anorectal abscess and undergoing incision and drainage were considered eligible for inclusion. Patients with a pre-existing fistula-in-ano, known Crohn’s disease or other inflammatory bowel disease, anorectal malignancy, tuberculosis, hidradenitis suppurativa, recurrent anorectal abscess, previous anorectal surgery, or those unwilling to participate were excluded from the study. At enrolment, clinical history and examination were used to identify previously diagnosed inflammatory bowel disease and features suggestive of secondary causes of anorectal sepsis. Occult inflammatory bowel disease could not be completely excluded in the absence of routine gastrointestinal investigations in all participants.

Sample Size Calculation

The sample size was calculated using the standard formula for estimation of a single proportion in a population:

$n = \frac{Z^2 \times P \times Q}{d^2}$, 

where n is the required sample size, Z is the standard normal deviate at 99% confidence (Z = 2.58), P is the assumed prevalence of fistula-in-ano following anorectal abscess drainage in the local setting (1% or 0.01), Q is 1 − P (0.99), and d is the absolute precision (5% or 0.05). 

Substituting the values, n = (2.58)² × 0.01 × 0.99/(0.05)² ≈ 26.4. The calculated minimum sample size was therefore approximately 27 patients, which was rounded up to 30 patients. The final sample size was increased to 65 consecutive eligible patients to improve the precision of the estimates and to provide greater representation across demographic, clinical, anatomical, and microbiological subgroups during the study period. 

Data collection

Baseline demographic characteristics, socioeconomic status, comorbidities, dietary pattern, smoking history, alcohol consumption, tobacco chewing, daily water intake, bowel habits, and relevant clinical history were recorded using a structured proforma. Clinical examination included documentation of abscess location, abscess type, abscess size, and other operative findings. Pus specimens obtained during incision and drainage were sent for microbiological culture and sensitivity testing. 

Follow-up and outcome assessment

Patients were reviewed at regular outpatient follow-up visits for six months. The primary outcome was the development of fistula-in-ano, diagnosed clinically by the presence of a persistent or recurrent external opening with discharge and confirmed by examination, with magnetic resonance imaging (MRI) fistulography or endoanal ultrasonography performed when clinically indicated. Patients who developed a fistula were further classified according to the Parks classification into intersphincteric, transsphincteric, suprasphincteric, and extrasphincteric fistulas. Intersphincteric fistulas are confined to the intersphincteric plane, transsphincteric fistulas cross the external anal sphincter, suprasphincteric fistulas pass above the puborectalis muscle before descending through the intersphincteric plane, and extrasphincteric fistulas pass from the rectum or anal canal through the pelvic floor and sphincter complex to the perianal skin without traversing the external anal sphincter [2].

Operational definitions

Dietary pattern was categorised as vegetarian or mixed diet according to the participant’s usual dietary practice. Fibre intake was estimated from the average self-reported daily consumption of fibre-rich foods and categorised as low (<2 servings/day), moderate (2-4 servings/day), or high (≥5 servings/day). Daily water intake was categorised as <1.5 L/day, 1.5-2.5 L/day, or >2.5 L/day. Fast-food consumption was classified as never, <1 time/week, 1-3 times/week, or >4 times/week based on self-reported dietary history. Constipation was defined as a self-reported history of infrequent bowel movements, passage of hard stools, or excessive straining during defecation. Fistulas developing during follow-up were classified according to the Parks classification as intersphincteric, transsphincteric, suprasphincteric, or extrasphincteric, based on their anatomical relationship to the anal sphincter complex and operative findings [2].

Statistical analysis

Data were entered into Microsoft Excel (Microsoft Corporation, Redmond, Washington, United States) and analysed using IBM SPSS Statistics for Windows, version 28.0 (IBM Corp., Armonk, New York, United States). Categorical variables were summarised as frequencies and percentages. Associations between categorical variables and fistula formation were assessed using the Pearson chi-square test. Fisher’s exact test or the Fisher-Freeman-Halton exact test with Monte Carlo estimation was used when expected cell frequencies were insufficient for Pearson chi-square analysis. No continuous variables were subjected to inferential comparison in the present analysis; variables such as abscess size were categorised a priori for analysis. Variables demonstrating statistically significant or clinically relevant associations on categorical analysis were further evaluated using univariate binary logistic regression where appropriate. Odds ratios (ORs) with 95% confidence intervals (CIs) were calculated. Socioeconomic status was excluded from logistic regression because no fistula events occurred in the upper socioeconomic group, resulting in complete separation and unstable parameter estimates. A two-sided p-value of <0.05 was considered statistically significant.

## Results

A total of 65 patients with anorectal abscess underwent incision and drainage and completed six months of follow-up. During the follow-up period, 22 patients (33.8%) developed fistula-in-ano, 39 patients (60.0%) demonstrated complete wound healing without fistula formation, and four patients (6.2%) developed recurrent anorectal abscess without evidence of fistula. 

Demographic characteristics

The demographic characteristics of the study participants are presented in Table 1. Of the 65 patients, 45 (69.2%) were male, and 20 (30.8%) were female. Fistula-in-ano developed in 20 of 45 male patients (44.4%) compared with two of 20 female patients (10.0%). Male sex demonstrated a statistically significant association with fistula formation (p = 0.007). The largest proportion of patients belonged to the age group of 41-50 years (n=19, 29.2%). Patients aged 61-70 years (n=14) had the highest proportion of fistula formation (n=9, 64.3%), developing fistula in ano. However, the overall association between age group and fistula formation was not statistically significant on Pearson chi-square testing (p = 0.094). 

**Table 1:** Demographic characteristics and association with fistula formation (N = 65) Pearson’s chi-square test was used. A two-sided p-value <0.05 was considered statistically significant.

Variable	No fistula (n=43), n (%)	Fistula (n=22), n (%)	Total, n (%)	Test statistic	p-value
Age group (years)					
18–30	11 (78.6)	3 (21.4)	14 (21.5)	χ² = 7.937	0.094
31–40	6 (75.0)	2 (25.0)	8 (12.3)		
41–50	13 (68.4)	6 (31.6)	19 (29.2)		
51–60	8 (80.0)	2 (20.0)	10 (15.4)		
61–70	5 (35.7)	9 (64.3)	14 (21.5)		
Sex					
Female	18 (90.0)	2 (10.0)	20 (30.8)	χ² = 7.337	0.007
Male	25 (55.6)	20 (44.4)	45 (69.2)		

Residential and socioeconomic characteristics

The residential and socioeconomic characteristics of the study population are presented in Table 2. Rural residents constituted 35 of the 65 patients (53.8%), whereas 30 patients (46.2%) were urban residents. Fistula-in-ano developed in 12 of 35 rural residents (34.3%) and 10 of 30 urban residents (33.3%), with no significant association between place of residence and fistula formation (p = 0.936). Most patients belonged to the middle socioeconomic group (n=30, 46.2%), followed by the lower socioeconomic group (n=24, 36.9%) and upper socioeconomic group (n=11, 16.9%). Fistula-in-ano developed in 13 of 24 patients (54.2%) in the lower socioeconomic group, nine of 30 patients (30.0%) in the middle socioeconomic group, and 0 of 11 patients (0.0%) in the upper socioeconomic group. Socioeconomic status was significantly associated with fistula formation on the Fisher-Freeman-Halton exact test (p = 0.003).

**Table 2:** Residential and socioeconomic characteristics Pearson’s chi-square test was used for place of residence. Fisher–Freeman–Halton exact test was used for socioeconomic status because of low expected cell frequencies (cell count <5). A two-sided p-value <0.05 was considered statistically significant.

Variable	Total (n=65), n (%)	Fistula (n=22), n (%)	No Fistula (n=43), n (%)	Statistical Test	p-value
Place of residence					
Rural	35 (53.8%)	12 (34.3%)	23 (65.7%)	χ² =0.007	0.936
Urban	30 (46.2%)	10 (33.3%)	20 (66.7%)		
Socioeconomic status					
Lower	24 (36.9%)	13 (54.2%)	11 (45.8%)	—	0.003
Middle	30 (46.2%)	9 (30.0%)	21 (70.0%)		
Upper	11 (16.9%)	0 (0.0%)	11 (100.0%)		

Dietary and bowel habit factors 

A mixed dietary pattern was reported by 27 of 65 patients (41.5%), of whom 13 (48.1%) developed fistula-in-ano, compared with nine of 38 patients (23.7%) following a vegetarian diet. Dietary pattern was significantly associated with fistula formation (p = 0.040). Daily water intake of less than 1.5 L was reported by 32 of 65 patients (49.2%), of whom 16 (50.0%) developed fistula-in-ano. Fistula formation occurred in five of 22 patients (22.7%) consuming 1.5-2.5 L/day and one of 11 patients (9.1%) consuming more than 2.5 L/day. Water intake was significantly associated with fistula formation (p = 0.019). 

Constipation was present in 40 of 65 patients (61.5%), among whom 19 (47.5%) developed fistula-in-ano, compared with 3 of 25 patients (12.0%) without constipation. Constipation was significantly associated with fistula formation (p = 0.003). Fibre intake and fast-food consumption were not significantly associated with fistula formation on categorical analysis (p = 0.077 and p = 0.122, respectively). The association between dietary and bowel habit factors and fistula formation is presented in Table 3.

**Table 3:** Dietary and bowel habits Pearson’s chi-square test; p < 0.05 was considered statistically significant.

Variable	No fistula (n=43), n (%)	Fistula (n=22), n (%)	Total (n=65), n (%)	Test statistic	P value
Diet					
Vegetarian	29 (76.3)	9 (23.7)	38 (58.5)	χ²= 4.219	0.040
Mixed	14 (51.9)	13 (48.1)	27 (41.5)		
Fast-food intake					
Never	7 (70.0)	3 (30.0)	10 (15.4)	χ²=5.794	0.122
<1 time/week	23 (74.2)	8 (25.8)	31 (47.7)		
1-3 times/week	12 (63.2)	7 (36.8)	19 (29.2)		
>4 times/week	1 (20.0)	4 (80.0)	5 (7.7)		
Fibre intake (servings/day)					
<2	17 (53.1)	15 (46.9)	32 (49.2)	χ² = 5.137	0.077
2–4	19 (76.0)	6 (24.0)	25 (38.5)		
≥5	7 (87.5)	1 (12.5)	8 (12.3)		
Water intake (L/day)					
<1.5	16 (50.0)	16 (50.0)	32 (49.2)	χ²= 7.955	0.019
1.5–2.5	17 (77.3)	5 (22.7)	22 (33.8)		
>2.5	10 (90.9)	1 (9.1)	11 (16.9)		
Constipation					
No	22 (88.0)	3 (12.0)	25 (38.5)	χ²=8.659	0.003
Yes	21 (52.5)	19 (47.5)	40 (61.5)		

Comorbidities and lifestyle factors

The association between comorbidities, personal habits, and fistula formation is presented in Table 4. Diabetes mellitus was the most common comorbidity, affecting 17 of 65 patients (26.2%), followed by obesity in 13 patients (20.0%) and hypertension in 11 patients (16.9%). Diabetes mellitus was significantly associated with fistula formation, with 10 of 17 diabetic patients (58.8%) developing fistula-in-ano (p=0.011). Alcohol consumption was the most frequently reported personal habit, occurring in 24 of 65 patients (36.9%), followed by smoking in 13 patients (20.0%) and tobacco chewing in nine patients (13.8%). None of these personal habits was significantly associated with fistula formation.

**Table 4:** Comorbidities and habits Pearson’s chi-square test was used where appropriate. Fisher’s exact test was used for variables with sparse expected cell frequencies. A two-sided p-value <0.05 was considered statistically significant. For binary variables, the p-value represents the overall comparison between the presence and absence of the respective condition or habit and is therefore reported once per variable rather than separately for each category.

Variable	Total (n=65), n (%)	Fistula (n=22), n (%)	No fistula (n=43), n (%)	Test Statistic	p-value
Comorbidities					
Diabetes mellitus	17 (26.2%)	10(58.8%)	7(41.2%)	χ^2 ^=6.414	0.011
Hypertension	11 (16.9%)	3 (27.3%)	8 (72.7%)	—	0.737
Obesity (BMI >30)	13(20%)	4 (30.8%)	9 (69.2%)	—	1.000
Immunocompromised	4 (6.2%)	2 (50.0%)	2 (50%)	—	0.599
Personal habits					
Smoking	13 (20%)	6 (46.2%)	7 (53.8%)	—	0.336
Alcohol consumption	24(36.9%)	7(29.2%)	17 (70.8%)	χ²= 0.372	0.542
Tobacco chewing	9 (13.8%)	1 (11.1%)	8 (88.9%)	—	0.152

Anatomical characteristics of abscesses

The anatomical characteristics of the anorectal abscesses and their association with subsequent fistula formation are presented in Table 5. Perianal abscess was the most frequently encountered abscess type, occurring in 42 of 65 patients (64.6%), followed by intersphincteric abscess in 16 patients (24.6%), ischiorectal abscess in four patients (6.2%), submucosal abscess in two patients (3.1%), and supralevator abscess in one patient (1.5%).

**Table 5:** Abscess characteristics Pearson’s chi-square test was used for abscess size. Fisher–Freeman–Halton exact test was used for abscess type and position because of sparse cell counts. A two-sided p-value <0.05 was considered statistically significant. *comprised ischiorectal, submucosal, and supralevator abscesses

Variable	No fistula (n=43), n (%)	Fistula (n=22), n (%)	Total (n=65), n (%)	Test statistic	p-value
Type of abscess				—	0.007
Perianal	32 (76.2)	10 (23.8)	42 (64.6)		
Intersphincteric	10 (62.5)	6 (37.5)	16 (24.6)		
Others*	1 (14.3)	6 (85.7)	7 (10.8)		
Position of abscess				—	0.013
Posterior	14 (46.7)	16 (53.3)	30 (46.2)		
Right lateral	9 (81.8)	2 (18.2)	11 (16.9)		
Left lateral	13 (76.5)	4 (23.5)	17 (26.2)		
Anterior	7 (100.0)	0 (0.0)	7 (10.8)		
Abscess size				χ² = 10.470	0.001
≤3 cm	26 (86.7)	4 (13.3)	30 (46.2)		
>3 cm	17 (48.6)	18 (51.4)	35 (53.8)		

Overall abscess type was significantly associated with fistula formation (Fisher-Freeman-Halton exact test, p = 0.007). Fistula developed in 10 of 42 patients (23.8%) with perianal abscesses, six of 16 patients (37.5%) with intersphincteric abscesses, and six of seven patients (85.7%) with other abscess types. Because of the small numbers in individual anatomical subgroups, abscess type was subsequently recoded for logistic regression as perianal abscess versus other abscess types. 

Abscess position was also significantly associated with fistula formation on categorical analysis (Fisher-Freeman-Halton exact test, p = 0.013). Posterior abscesses demonstrated the highest proportion of fistula formation, with 16 of 30 patients (53.3%) developing fistula-in-ano, compared with two of 11 patients (18.2%) with right lateral abscesses, four of 17 patients (23.5%) with left lateral abscesses, and none of the seven patients (0.0%) with anterior abscesses. Abscess size was significantly associated with fistula formation. Among the 35 patients with abscesses measuring greater than 3 cm, 18 (51.4%) developed fistula-in-ano compared with four of 30 patients (13.3%) with abscesses measuring 3 cm or less (χ² = 10.470, p = 0.001). 

Anaesthesia and postoperative treatment

Spinal anaesthesia was used in 63 patients (96.9%), while general anaesthesia was used in two patients (3.1%). The type of anaesthesia was not significantly associated with fistula formation (p=0.741). All 65 patients received postoperative antibiotics according to institutional protocol. As there was no non-antibiotic comparison group, the independent effect of postoperative antibiotics on fistula prevention could not be evaluated.

Microbiological profile

*Escherichia coli* was the most frequently isolated organism, identified in 22 of 65 patients (33.8%), followed by mixed bacterial growth in 13 patients (20.0%), *Klebsiella pneumoniae* in 11 patients (16.9%), and *Staphylococcus aureus* in six patients (9.2%). Cultures were sterile in nine patients (13.8%), while culture was not performed in four patients (6.2%).

Incidence and outcome at six months

At six months, 22 patients (33.8%) developed fistula-in-ano, 39 (60.0%) achieved complete wound healing without fistula, and four (6.2%) developed recurrent anorectal abscess without fistula (Table 6).

**Table 6:** Clinical outcomes at six-month follow-up after incision and drainage of anorectal abscess.

Outcome at six-month follow-up	Number of patients	Percentage (%)
Fistula-in-ano developed	22	33.8%
Complete healing (no fistula)	39	60.0%
Recurrent abscess without fistula	4	6.2%
Total	65	100%

Of the 22 patients who developed fistula-in-ano, 16 (72.7%) were diagnosed within the first three months, while the remaining six (27.3%) developed fistula between three and six months. Thus, 72.7% of all fistulas had developed by three months and 100% by six months, as depicted in Table 7.

**Table 7:** Time of development of fistula-in-ano

Follow-up period	New fistulas diagnosed, n	Fistulas developed, n (%)
1 month	3	3 (13.6%)
2 months	7	10 (45.5%)
3 months	6	16 (72.7%)
6 months	6	22 (100%)

Characteristics of established fistulas

The anatomical characteristics of the 22 established fistulas are presented in Table 8. A posterior internal opening was the most frequent finding, occurring in 13 of 22 patients (59.1%), followed by anterior openings in four patients (18.2%), left lateral openings in three patients (13.6%), and right lateral openings in two patients (9.1%). According to the Parks classification, intersphincteric fistulas were the most common subtype, occurring in 16 of 22 patients (72.7%), followed by transsphincteric fistulas in four patients (18.2%). Suprasphincteric and extrasphincteric fistulas each occurred in one patient (4.5%). Single external openings were present in 15 patients (68.2%), whereas seven patients (31.8%) had multiple external openings.

**Table 8:** Anatomical characteristics of fistula-in-ano among patients who developed fistula (N = 22)

Characteristics of fistula-in-ano	Frequency	Percentage
Location of internal opening		
Posterior	13	59.1
Anterior	4	18.2
Left lateral	3	13.6
Right lateral	2	9.1
Parks classification		
Intersphincteric	16	72.7
Transsphincteric	4	18.2
Suprasphincteric	1	4.5
Extrasphincteric	1	4.5
External openings		
Single external opening	15	68.2
Multiple external openings	7	31.8

Univariate binary logistic regression analysis

The results of the univariate binary logistic regression analysis are presented in Table 9. The analysis included 65 patients, comprising 22 patients who developed fistula-in-ano and 43 patients who did not develop fistula-in-ano. 

**Table 9:** Univariate logistic regression analysis of factors associated with fistula formation following incision and drainage of anorectal abscess (n = 65) Reference categories: Female sex; age 18–30 years; abscess size ≤3 cm; vegetarian diet; absence of constipation; absence of diabetes mellitus; Daily water intake 1.5–2.5 L/day; and perianal abscess Socioeconomic status was excluded from logistic regression because no fistula events occurred in the upper socioeconomic group, resulting in unstable parameter estimates and complete separation.

Risk factor	OR (95% CI)	p-value
Male sex	7.20 (1.36–38.20)	0.014
Age 61–70 years	6.60 (1.23–35.44)	0.028
Abscess size >3 cm	6.88 (1.98–23.88)	0.002
Mixed diet	2.99 (1.03–8.66)	0.043
Constipation	6.64 (1.71–25.76)	0.006
Diabetes mellitus	4.28 (1.33–13.75)	0.014
Daily water intake <1.5 L	3.40 (1.01–11.45)	0.048
Other abscesses (ischiorectal, submucosal, supralevator) vs perianal abscess	19.20 (2.06–179.08)	0.009

Male sex was associated with significantly increased odds of fistula formation compared with female sex (OR 7.20, 95% CI 1.36-38.20; p = 0.014). Patients aged 61-70 years had significantly higher odds of fistula formation compared with those aged 18-30 years (OR 6.60, 95% CI 1.23-35.44; p = 0.028). Abscess size greater than 3 cm was associated with significantly increased odds of fistula formation compared with abscesses measuring 3 cm or less (OR 6.88, 95% CI 1.98-23.88; p = 0.002). 

A mixed dietary pattern was associated with increased odds of fistula formation compared with a vegetarian diet (OR 2.99, 95% CI 1.03-8.66; p = 0.043). Constipation was associated with increased odds of fistula formation (OR 6.64, 95% CI 1.71-25.76; p = 0.006). Diabetes mellitus was also associated with increased odds of fistula formation (OR 4.28, 95% CI 1.33-13.75; p = 0.014). Daily water intake of less than 1.5 L was associated with increased odds of fistula formation compared with 1.5-2.5 L/day (OR 3.40, 95% CI 1.01-11.45; p = 0.048).

Patients with other abscess types, comprising ischiorectal, submucosal, and supralevator abscesses, had significantly higher odds of fistula formation compared with patients with perianal abscesses (OR 19.20, 95% CI 2.06-179.08; p = 0.009). Although posterior abscess location was significantly associated with fistula formation on categorical analysis (p = 0.013), it was not statistically significant on univariate logistic regression (p = 0.113) and was therefore not considered a significant regression predictor. 

Socioeconomic status was not included in the logistic regression analysis because no fistula events occurred in the upper socioeconomic group, resulting in complete separation and unstable parameter estimates.

## Discussion

The present prospective study found that the six-month cumulative incidence of fistula-in-ano following incision and drainage of anorectal abscess was 33.8%. This finding is consistent with previously reported studies. Henrichsen and Christiansen reported a fistula rate of 26% following anorectal sepsis [4], while Hämäläinen and Sainio reported a rate of 37% [5], and Hasan et al. reported a rate of 33.9% following abscess drainage [6]. Similar rates have also been reported in more recent observational studies [7-10]. Differences between studies may reflect variations in patient characteristics, abscess characteristics, follow-up duration, and methods of outcome assessment.

Male sex demonstrated a significant association with fistula formation. Fistula-in-ano developed in 20 of 45 males (44.4%) compared with two of 20 females (10.0%). On univariate logistic regression, male patients had significantly higher odds of developing fistula-in-ano compared with females (OR 7.20, 95% CI 1.36-38.20; p=0.014). The wide CI reflects the limited precision associated with the relatively small sample size.

Age group was not significantly associated with fistula formation on overall Pearson chi-square analysis (p=0.094). However, patients aged 61-70 years had the highest proportion of fistula formation (9/14, 64.3%) and significantly higher odds compared with those aged 18-30 years on univariate logistic regression (OR 6.60, 95% CI 1.23-35.44; p=0.028). This finding should be interpreted cautiously because of the small number of patients in individual age categories and the lack of statistical significance in the overall age-group comparison. It should therefore be considered hypothesis-generating rather than definitive.

Socioeconomic status demonstrated a statistically significant association with fistula formation. Patients belonging to the lower socioeconomic group had the highest proportion of fistula formation (54.2%), followed by the middle socioeconomic group (30.0%), whereas no fistula occurred among patients in the upper socioeconomic group. The overall association was significant on Fisher-Freeman-Halton exact testing (p=0.003). Lower socioeconomic status may reflect differences in nutrition, personal hygiene, health literacy, healthcare-seeking behaviour, and access to timely medical care, which may potentially influence postoperative recovery and persistent infection. However, socioeconomic status was not included in logistic regression because no fistula events occurred in the upper socioeconomic group, resulting in complete separation and unstable parameter estimates. Therefore, an adjusted or regression-derived odds ratio should not be interpreted for socioeconomic status in the present study.

Anatomical characteristics of the abscess demonstrated significant associations with fistula formation. Overall abscess type was significantly associated with fistula formation on Fisher-Freeman-Halton exact testing (p=0.007). Following recoding because of the small numbers in individual anatomical subgroups, patients with ischiorectal, submucosal, or supralevator abscesses had significantly higher odds of fistula formation compared with those with perianal abscesses (OR 19.20, 95% CI 2.06-179.08; p=0.009). The wide confidence interval indicates considerable uncertainty around this estimate and is likely related to the small number of patients in the individual non-perianal subgroups.

Abscess position was also significantly associated with fistula formation on categorical analysis (p=0.013), with posterior abscesses demonstrating the highest proportion of fistula formation. However, posterior abscess location was not statistically significant on univariate logistic regression (p=0.113). Therefore, although overall abscess position was associated with fistula formation, the present study does not establish posterior location as a significant regression predictor.

Abscess size demonstrated a significant association with fistula formation. Patients with abscesses >3 cm had significantly higher odds of fistula formation than those with abscesses ≤3 cm (OR 6.88, 95% CI 1.98-23.88; p=0.002). Larger abscesses may reflect a greater extent of initial infection and tissue involvement and may therefore be associated with persistent sepsis or subsequent fistulisation [8,9].

Several lifestyle-related factors demonstrated significant associations with fistula formation. Patients consuming a mixed diet demonstrated a significantly higher proportion of fistula formation than those following a vegetarian diet (48.1% vs 23.7%; p=0.040), and a mixed dietary pattern was associated with increased odds of fistula formation on univariate logistic regression (OR 2.99, 95% CI 1.03-8.66; p=0.043). Similarly, daily water intake below 1.5 L was significantly associated with fistula formation (p=0.019), with patients consuming less than 1.5 L/day demonstrating increased odds on logistic regression (OR 3.40, 95% CI 1.01-11.45; p=0.048). These findings are potentially clinically relevant because dietary and hydration practices are modifiable factors. However, they should be interpreted cautiously because dietary pattern and hydration may be interrelated with other behavioural and socioeconomic factors.

Constipation was significantly associated with fistula formation (p=0.003) and was associated with increased odds on univariate logistic regression (OR 6.64, 95% CI 1.71-25.76; p=0.006). The association may be clinically relevant because constipation and straining are potentially modifiable factors. Nevertheless, the present observational study cannot establish whether constipation directly contributes to fistula development or represents a marker of other underlying dietary or lifestyle characteristics.

Diabetes mellitus was significantly associated with fistula formation (p=0.011). On univariate logistic regression, diabetes mellitus was associated with significantly increased odds of fistula formation (OR 4.28, 95% CI 1.33-13.75; p=0.014). Impaired immune function, altered tissue perfusion, and delayed wound healing may contribute to persistent infection in patients with diabetes, although the present study cannot establish causality. Smoking, alcohol consumption, and tobacco chewing were not significantly associated with fistula formation. Similarly, fibre intake did not demonstrate a statistically significant association with fistula formation (p=0.077).

Microbiological findings demonstrated that *E. coli* was the most frequently isolated organism (33.8%), followed by mixed bacterial growth (20.0%) and *K. pneumoniae* (16.9%). However, inferential statistical analysis of individual organisms was not performed because of the small numbers within individual microbiological categories. Therefore, the present study cannot determine whether specific microorganisms independently predict fistula formation. As all patients received postoperative antibiotics according to institutional protocol, their independent effect on fistula prevention could not be evaluated.

Accurate anatomical assessment of fistula-in-ano may require imaging in selected patients, as clinical examination alone may not fully delineate the fistulous tract; endoanal ultrasonography and MRI can provide complementary anatomical information [11].

Among patients who developed fistula-in-ano, posterior internal openings were the most frequent, identified in 13 of 22 patients (59.1%). According to the Parks classification, intersphincteric fistulas were the predominant subtype, occurring in 16 of 22 patients (72.7%), followed by transsphincteric fistulas (18.2%), suprasphincteric fistulas (4.5%), and extrasphincteric fistulas (4.5%). This distribution is compatible with established patterns of cryptoglandular fistula disease and previous clinical series [12-14]. These findings describe the anatomical characteristics of established fistulas and should not be interpreted as predictors of fistula development.

Overall, the findings support structured clinical surveillance following anorectal abscess drainage, particularly among patients with larger abscesses and those with demographic, dietary, hydration, bowel habit, comorbidity, socioeconomic, and anatomical characteristics that were associated with fistula formation in the present cohort. However, these associations should be regarded as hypothesis-generating rather than causal or independently predictive. Larger prospective multicentre studies are required to validate these findings.

Strengths and limitations

The present study has several strengths. It prospectively assessed patients following incision and drainage of anorectal abscess, with structured six-month follow-up for identification of fistula formation and recurrent abscess. The study evaluated a broad range of demographic, socioeconomic, clinical, anatomical, microbiological, dietary, and lifestyle-related variables. Detailed assessment of abscess size, location, type, and the anatomical characteristics of established fistulas further strengthens the clinical description of the cohort.

Several limitations should be acknowledged. The relatively small sample size and single-centre design limit generalisability. The six-month follow-up may not detect late fistula formation. Dietary, hydration, and bowel habit data were self-reported and therefore subject to recall and reporting bias. The small numbers in some abscess and socioeconomic subgroups limited statistical precision and resulted in wide confidence intervals for some estimates. MRI or endoanal ultrasonography was not routinely performed in all patients; therefore, occult or anatomically complex fistulas may have been under-recognised. Finally, multivariable logistic regression was not performed, and therefore the observed associations should not be interpreted as independent predictors of fistula formation.

## Conclusions

In this prospective cohort, 22 of 65 patients (33.8%) developed fistula-in-ano within six months following incision and drainage of anorectal abscess. Male sex, age 61-70 years, larger abscess size, mixed dietary pattern, constipation, diabetes mellitus, low daily water intake, and ischiorectal, submucosal, and supralevator abscesses were associated with increased odds of fistula formation on univariate logistic regression. Lower socioeconomic status and overall abscess position were significantly associated with fistula formation on categorical analysis, although specific regression associations could not be established. These findings highlight clinical and potentially modifiable characteristics that may help identify patients who require closer follow-up after abscess drainage. However, given the small single-centre cohort and absence of multivariable analysis, these associations should not be considered independent predictors or causal relationships. Larger multicentre prospective studies with longer follow-up and multivariable analysis are required to validate these findings and identify independent predictors of fistula formation.
